# List of Hard Ticks (Acari: Ixodida: Ixodidae) in Subterranean Habitats in Croatia

**DOI:** 10.3390/pathogens15030343

**Published:** 2026-03-23

**Authors:** Stjepan Krčmar, Roman Ozimec

**Affiliations:** 1 Department of Biology, Josip Juraj Strossmayer University of Osijek, Cara Hadrijana 8/A, 31000 Osijek, Croatia; 2 ADIPA: Society for Research & Conservation of Croatian Natural Diversity, Orehovečki Ogranak 37, 10040 Zagreb, Croatia; roman.ozimec@adipa.hr

**Keywords:** hard ticks, Ixodidae, subterranean habitats, Croatia, taxonomy, biogeography

## Abstract

Between 1993 and 2024, a total of 274 hard ticks (Ixodidae) were collected from 138 subterranean localities in Croatia. This study represents the most extensive survey of hard tick fauna in subterranean habitats in Croatia to date. The collected specimens were classified into three genera and seven taxa, including two taxa that could not be identified to the species level (one from the genus *Ixodes* and one from *Haemaphysalis*). The genus *Ixodes* was the most abundant, comprising five taxa, whereas *Haemaphysalis* and *Hyalomma* were each represented by a single taxon. The highest diversity of hard ticks was recorded in subterranean habitats in Dalmatia, followed by north-western Croatia and Slavonia. *Ixodes vespertilionis* Koch, 1844 was the dominant species in the collected sample, representing 81.0% of all specimens, and was recorded in all studied regions. This species was present throughout the entire year, whereas *I. hexagonus* Leach, 1815 was recorded during nine months, *I. frontalis* (Panzer, 1798) during four months, and the remaining taxa during shorter periods. The largest number of *I. vespertilionis* specimens was collected in spring (33.2%), while the lowest number was recorded in winter (16.6%). The record of *I. frontalis* represents the first documented occurrence of this species in subterranean habitats in Croatia.

## 1. Introduction

Hard ticks (Ixodidae) are obligatory hematophagous ectoparasites of many vertebrate species from the classes Mammalia, Aves, Reptilia and Amphibia [1], and can transmit various bacteria, viruses, parasitic protozoa and helminths to a wide range of domestic and wild animals from these classes, including humans [2,3,4]. Their blood-feeding habit and the exploitation of different hosts during the larval, nymphal, and adult stages increase the vector potential of ticks, making them the second most important vectors of disease agents in animals and humans after mosquitoes [5]. Due to their high vector capacity, hard ticks have been intensively studied worldwide in various natural ecosystems [6], as well as in numerous anthropogenic habitats such as urban green spaces and various agroecosystems [7,8,9,10]. Among the great diversity of existing biotopes, subterranean habitats (caves, semi-caves, and pits) are notable for their specific microclimate, lighting conditions, and the unique nature of their connections with other terrestrial ecosystems [11].

Although subterranean habitats in Central Europe host important vector groups of arthropods, such as hard ticks, taxonomically focused and geographically widespread studies in these habitats remain rare [12]. In Croatia, studies on the diversity of hard tick fauna and their vector potential in various natural ecosystems and anthropogenic habitats began in the first half of the last century and have continued to the present day. This is confirmed by recent findings of several species of the genus *Ixodes* newly recorded in the Croatian fauna [13,14]. In Croatia, hard ticks have mainly been collected using the flag-dragging method over vegetation in different ecosystems and manually from live or dead animals. However, data on the fauna of hard ticks from subterranean habitats remain very scarce. The first cavernicolous hard tick recorded in Croatia was *Ixodes vespertilionis* (reported as *Eschatocephalus gracilipes*), found in the cave Trojama u Srijanima in Dalmatia by Umberto Girometta, a gymnasium professor and speleologist from Split [15]. The university professor and entomologist August Langhoffer from Zagreb later confirmed Girometta’s finding and also reported the same taxon from a cave on the island of Vis (Špilja od Vore), from the Varaždin region (Repnjak), and from Medvednica Mt. (Žurenščak) [16]. The most important contribution to the knowledge of the tick fauna of the Dinarides was provided by material collected by the renowned Moravian biospeleologist Karel Absolon before the Second World War. This material was investigated by the German acarologist Carl Willmann, initially in short papers [17,18], and later in the monograph Die Acari der Höhlen der Balkanhalbinsel [19]. In this monograph, four taxa were reported for Croatia, partly determined by the German acarologist Paul Leopold Ernst Schulze: *Ixodes vespertilionis* from seven caves, *Ixodes ricinus* from one cave on Mljet Island, *Ixodes hexagonus* from Vrlovka Cave near Kamanje, and *Hyalomma marginatum* from one cave in Istria [19]. This contribution represents the most extensive dataset on hard tick fauna collected in subterranean habitats in Croatia since the Second World War. The aim of this study was to improve knowledge of the diversity of hard tick fauna in subterranean habitats in Croatia and, for the first time, to analyse the abundance, distribution, and seasonal dynamics of the recorded taxa.

## 2. Materials and Methods

### 2.1. Study Area

#### 2.1.1. Croatian Dinaric Karst, Caves, and Cave Habitats

Croatia is a southern European country located in the Mediterranean, Pannonian, Dinaric, and Balkan regions, and is a member of the European Union. Karst covers approximately 26,000 km^2^ of southern Croatia, including the islands. The territory of Croatia includes four major macrotectonic units: the Adriatic, Dinaric, Supradinaric, and Pannonian [20]. The Dinarides occupy about 46% of Croatia’s territory and account for approximately 40% of the total surface area of the Dinarides (65,000 km^2^) [21].

More than 10,000 caves have been recorded in the Dinaric part of Croatia, and it is estimated that at least the same number remain undiscovered. In other, non-Dinaric regions of Croatia, only small areas of isolated karst or non-karstic caves occur. Thirty-one caves are protected under nature conservation legislation: 29 are classified as geomorphological monuments of nature and two as palaeontological sites [20]. More than 300 caves are protected as Natura 2000 sites, and many others are included within larger protected Natura 2000 areas. According to the Croatian National Habitat Classification, harmonised with the European Habitats Directive, the most important cave habitats where hard ticks may occur include karstic caves and pits, terrestrial karstic cave habitats, and caves inhabited by subtroglophilic vertebrates. The most important vertebrates in these cave habitats are bats (Chiroptera) and their associated parasitic and guanophilic organisms [20].

#### 2.1.2. Cave Biodiversity

Biospeleological research in Croatia began in the first half of the 19th century, at the very inception of biospeleology, and has continued to the present day with an increasing number of scientists involved. Practically all taxonomic kingdoms can be found in cave habitats; however, photosynthetic flora occurs only in illuminated entrance zones, and, as in surface habitats, fauna predominates. The total number of cave taxa (stygobionts and troglobionts) recorded for Croatia and estimated up to 2002 is approximately 469 taxa [22]. In addition to cave-adapted taxa, numerous troglophilic and stygophilic taxa occur, including edaphic, guanophilic, and parasitic organisms, among which hard ticks (Ixodidae) are included.

### 2.2. Samplings and Identification

#### 2.2.1. Fieldwork Methodology

**Figure 1:**
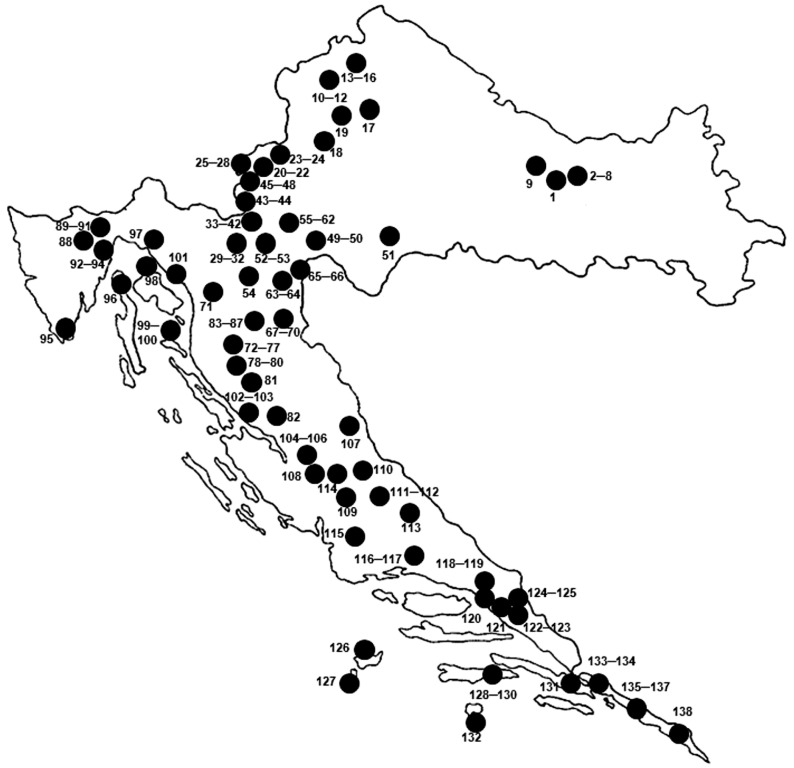
Sampling localities of hard ticks in subterranean habitats in Croatia, as listed in Appendix A.

All hard ticks were collected during systematic biospeleological research conducted in caves at 138 locations. The sampling was carried out or organised primarily by the second author, or the material was donated by colleagues (Appendix A, Figure 1).

The research included the identification of cave habitats, microclimatic measurements (air temperature, sediment temperature, and water temperature where present; relative air humidity; and CO_2_ content), assessment of cave biodiversity, and the collection of specimens from selected taxonomic groups or soil and water samples. Sampling for hard ticks was usually performed after visual observation, using tweezers to collect specimens from cave walls, stone crevices, or sinter formations. Typically, only the presence of bats or other mammals or vertebrates was noted, together with the number of individuals observed. It should be noted that in many cases no vertebrates were observed; therefore, the presence of ticks indicates the periodic presence of bats or other mammals, which was also inferred from observed excrements. In this way, in addition to bats, the presence of the European edible dormouse (*Glis glis*) and martens (*Martes* sp.) was most frequently documented. As part of the fieldwork methodology, many tick specimens were photographed in situ. All collected specimens were preserved in 8 mL plastic vials containing 70% ethanol. After collection, the vials were labelled with a hard paper label containing the minimum necessary handwritten information: cave name, basic location, date of sampling, and collector names. The material was then deposited in the Roman Ozimec Collection (ROC) for further processing.

#### 2.2.2. Tick Identification

The identification of the collected hard ticks (sex and species) was performed using a Carl Zeiss Jena stereomicroscope (×40 magnification) according to available identification keys [23,24,25] and illustrated guides for tick identification [26,27]. For each record, the following data are provided: species name, locality, main locality (village, city, mountain, or protected area), name of municipality or city, or name of island, date of collection, name of collector, number and sex of specimens, and depository (Appendix B). The survey localities are marked with a running number referring to the map in Figure 1, and the UTM grid (10 × 10 km) is given in the third column of Appendix A, following the system used in the Catalogue of Cave Type Localities of Croatian Fauna [28]. Geographic coordinates are listed only for the first toponym (Appendix A), as exact coordinates for subterranean localities cannot be provided due to the protection of these speleological sites. This is in accordance with the instructions of the Ministry of Environmental Protection and Green Transition of the Republic of Croatia regarding the protection of such sites. All hard ticks from the tick collections (ROC) are deposited in the Natural Science Department of the Varaždin City Museum (GMV).

## 3. Results

A total of 274 specimens of hard ticks (Ixodidae) were collected from 138 subterranean habitats located in 13 regions of Croatia (Table A1 and Table A2; Appendix A and Appendix B). The collected ticks were classified into three genera and seven taxa (Table 1).

**Figure 2:**
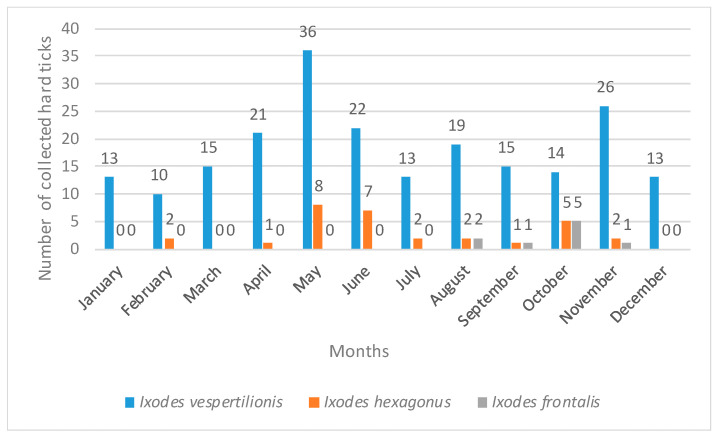
Seasonal activity patterns of the three dominant hard tick (Ixodidae) species in the study area.

The most abundant genus was *Ixodes* Latreille, 1795, represented by five taxa, whereas the other two genera, *Haemaphysalis* Koch, 1844 and *Hyalomma* Koch, 1844, were each represented by a single taxon (Table 2). *Ixodes vespertilionis* Koch, 1844 was the most abundant species in the collected sample, followed by *Ixodes hexagonus* Leach, 1815, *Ixodes frontalis* (Panzer, 1798), *Ixodes ricinus* (Linnaeus, 1758), *Ixodes* sp., *Haemaphysalis* sp., and *Hyalomma marginatum* Koch, 1844 (Table 2). The majority of collected ticks (93.8%) were adults, whereas the nymphal stage accounted for 6.2% of specimens. Males represented 76.2% of the collected sample, females 17.5%, and nymphs 6.2% (Table 2). Nymphs were recorded only in the species *I. vespertilionis*. The largest number of hard ticks (52 specimens) was recorded in Pokuplje and Dalmatia. However, the highest number of taxa (five) was recorded in Dalmatia. Four taxa of hard ticks were observed in north-western Croatia (Croatian Zagorje and the Varaždin area), three in Slavonia, whereas two or one species were recorded in other regions (Table 2). Only *I. vespertilionis* was recorded in all 13 studied regions of Croatia. *I. hexagonus* was recorded in seven Croatian regions, followed by *I. frontalis* in three regions, while the remaining four taxa were recorded in only one region each (Table 2). *Ixodes vespertilionis* was recorded at 112 subterranean localities, appearing at 81.1% of the studied localities. It is followed by *I. hexagonus* at 21 localities (15.2%) and *I. frontalis* at seven localities (5.0%), whereas other hard tick taxa were recorded at a smaller number of localities (Appendix B). In Slavonia and Croatian Zagorje, *I. vespertilionis* was recorded at 33.3% of the studied localities, whereas in other regions it was present at 70% to 100% of the localities (Appendix B). The record of *I. frontalis* in subterranean localities in Slavonia, Istria, and Dalmatia represents a new record for the subterranean fauna of Croatia (Table 2; Appendix B). Only one species, *I. vespertilionis*, was recorded during all 12 months of the year (Figure 2).

*I. hexagonus* was recorded during nine months of the year, *I. frontalis* during four months, and the other taxa were collected over a shorter period of one or two months. The highest activity of *I. vespertilionis* was recorded in May and November, with 28.6% of specimens collected during these two months (Figure 2). However, the largest number of *I. vespertilionis* specimens was collected during the spring months (33.2%), while the smallest number was recorded in winter (16.6%). During the summer and autumn months, nearly equal numbers of *I. vespertilionis* specimens were collected (24.9% and 25.3%, respectively) (Figure 2). The highest activity of *I. hexagonus* was recorded in May and June, with considerably lower numbers in other months, except in October (Figure 2). *I. hexagonus* and *I. frontalis*, the second and third most abundant species, differed significantly in their seasonal patterns. The highest abundance of *I. hexagonus* was recorded in summer (36.7%), whereas the highest abundance of *I. frontalis* was recorded in autumn (77.8%) (Figure 2). Seasonality of the remaining four taxa was not analysed due to the significantly smaller number of collected specimens. Of the seven recorded tick taxa, *I. hexagonus* and *I. vespertilionis* were recorded in all three biogeographic regions of Croatia (Continental, Alpine, and Mediterranean). *I. frontalis* was recorded in two biogeographic regions (Continental and Mediterranean), whereas the remaining taxa were recorded in only one biogeographic region (Table 2).

## 4. Discussion

**Figure 3:**
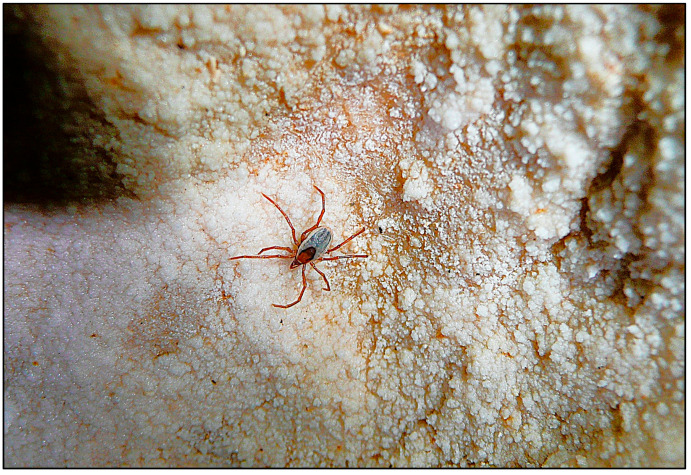
Female *Ixodes vespertilionis* observed in situ in Biserujka Cave, Krk Island, 26 May 2009. Photo by R. Ozimec.

**Figure 4:**
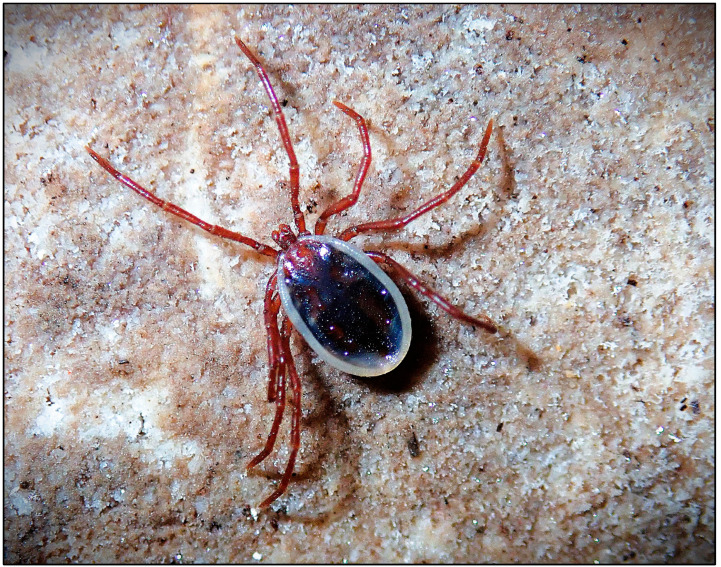
Male *Ixodes vespertilionis* observed in situ in Pišurka Cave, Korčula Island, 4 December 2018. Photo by R. Ozimec.

Comparison of the hard tick fauna recorded in this study with those reported from other European studies indicates that subterranean habitats in Croatia host a very similar tick fauna. Six species of hard ticks have been recorded in cave habitats in the Central German uplands and Luxembourg [12], of which two species, *I. hexagonus* and *I. ricinus*, were also recorded in this study. Furthermore, five species of hard ticks have been reported from cave habitats in Turkey [1], including *I. vespertilionis*, which was also recorded in this study. In Crimea and the Caucasus region of Russia, four species of hard ticks have been recorded from caves [11]; three of these (*I. vespertilionis*, *I. hexagonus*, and *H. marginatum*) were also found in this study. Three nidicolous species of hard ticks have been recorded in Central Europe, specifically associated with bats [29], of which only *I. vespertilionis* was recorded in the present study, as the other two species have not yet been documented in Croatia. Only *I. vespertilionis* (Figure 3 and Figure 4) was found in all studied regions of Croatia and was collected from cave walls and crevices by speleologists. Outside caves and cave-like shelters, *I. vespertilionis* occurs exclusively attached to its hosts [30]. In this study, no specimens were collected directly from bats, although numerous studies report this species on a wide variety of bat hosts at both immature and adult stages [31]. Studies conducted in Slovenia, Hungary, and Turkey indicate that *I. vespertilionis* is the most prevalent species in caves, occurring primarily on walls and in crevices [1,32,33]. In Slovenia, most *I. vespertilionis* were collected from January to April [32], which partially coincides with our data, as in Croatia the largest numbers of *I. vespertilionis* were collected in spring and autumn. Two peaks of abundance were observed: the first in May and the second in November (Figure 2). A similar seasonal pattern was reported in Hungary, with maximum abundance in spring [33]. During spring, pregnant female bats arrive at caves in groups of up to several hundred individuals, forming nursery colonies [30,34,35]. The first peak of *I. vespertilionis* abundance in May is consistent with this behavior, as the formation of nursery colonies provides an adaptive advantage for parasites to maximize feeding and reproductive success [35]. Female bats form small or large aggregations during the nursing period, making them particularly accessible to ticks [35]. Females remain in these caves until September to give birth and raise their young [30]. This pattern of host behavior likely explains the continued presence of substantial numbers of *I. vespertilionis* in caves throughout the summer. The second peak in November is probably related to bat colonies gathering in winter roosts, as hibernation typically occurs from late October to late March [34]. In Slavonia and Croatian Zagorje, *I. vespertilionis* was recorded at only 33.3% of studied localities, mainly during nursing and mating periods, whereas in other regions it was present at 70% to 100% of localities (Appendix B). Previous studies reported sporadic records of *I. vespertilionis* in Croatia from only a few localities: Trojama u Srijanima [15]; Špilja od Vore, Vis Island; Varaždin region (Repnjak); Medvednica Mt. (Žurenščak) [16]; Močiljska špilja (Dubrovnik); Petrićevi špilja, Čampari (Cres Island); Rabakova peć, Ročko polje (Roč); and Krbavsko polje [32,36]. The present study confirms these earlier findings in three cave habitats (Petrićevi špilja, Čampari, Cres Island; Rabakova peć, Ročko polje, Roč; Špilja [rudnik] od Vore, Vis Island). *I. vespertilionis* and *I. hexagonus* often occupy the same type of subterranean habitat, but they utilize different hosts [32]. In the present study, both species were collected together in only four of the 138 investigated subterranean localities (Appendix B). These localities were in north-western Croatia (Varaždin area: caves Zdenac pri Ciglaru, Klenovnik, Ravna Gora Mt.), Pokuplje (caves Pivnica jama, Sela Žakanjska, Hrenov Grič, Kamanje), and Kordun (cave Dragina peć na Dobri, Gabrk, Podumol) (Appendix B). In the western Palearctic, free-living stages of *I. vespertilionis* predominate in caves, with males recorded more frequently than females [37]. A similar pattern was observed in this study, where males represented 78.8% of specimens. This sex ratio may be explained by the fact that males do not feed and may live longer than females, which die after laying eggs [37]. The distribution of *I. vespertilionis* in the western Palearctic overlaps with the geographic ranges of five primary bat hosts: the greater mouse-eared bat (*Myotis myotis*), Maghrebian mouse-eared bat (*M. punicus*), Mediterranean horseshoe bat (*Rhinolophus euryale*), greater horseshoe bat (*R. ferrumequinum*), and lesser horseshoe bat (*R. hipposideros*) [37]. Four of these five species have been recorded in the Croatian fauna [38]. Although *R. hipposideros*, *R. ferrumequinum*, and *M. myotis* are considered the preferred hosts in Europe [37,39], this distribution explains why *I. vespertilionis* has been recorded in all studied regions. In Slavonia, hosts for *I. vespertilionis* are *R. hipposideros* and *M. myotis*, whereas in other studied areas *R. ferrumequinum* and *M. myotis* are the most likely hosts [38]. Of all hard ticks recorded in this study, only *I. vespertilionis* was recorded throughout the year. This pattern has also been observed in Slovenia and Hungary [32,33] and is likely a consequence of the stable microclimatic conditions in caves, which allow *I. vespertilionis* to remain active year-round [30].

*I. hexagonus* was the second most abundant species, representing 10.9% of ticks, and was recorded in seven of the 13 surveyed Croatian regions. This hard tick has previously been documented in subterranean habitats in Croatia, having been collected in the early 20th century from Vrlovka Cave, Kamanje in Pokuplje [19], a finding corroborated in the present study. *I. hexagonus* primarily inhabits lowlands but also occurs in mountainous regions up to 1000 m a.s.l. [40]. In these areas, it is ecologically restricted to caves [40], as confirmed by records from Papuk Mountain (953 m a.s.l.) in Slavonia, northwestern Croatia (Ivanščica 1059 m, Ravna Gora 686 m, Strahinjčica 846 m), and Velebit (1214–1757 m). In the study area, *I. hexagonus* was recorded in nine months, whereas *I. vespertilionis* was observed year-round. This reflects their endophilic lifestyles: one as a nest parasite associated with host burrows and dens, the other as a cave-dwelling species [34,41,42]. Subterranean habitats provide stable microclimatic conditions favoring free-living stages, independent of external weather [30,32,40]. Hedgehogs (*Erinaceus roumanicus*), a primary host of *I. hexagonus*, often hibernate in semi-caves or at cave entrances [40]. Other hosts from Mustelidae and Canidae families also frequent these habitats [40]. Occasionally, *I. hexagonus* has been recorded on bats such as *M. myotis* and *R. ferrumequinum* [37]. *I. frontalis*, the third most abundant species (3.3%), was recorded in cave habitats of Papuk (Slavonia), Učka (Istria), and Biokovo (Dalmatia), representing a new subterranean record for Croatia. In Dubrovnik, it was recorded on Bohemian waxwing (*Bombycilla garrulus*) and black redstart (*Phoenicurus ochruros*) [43]. *I. frontalis* is an ornithophilic tick, infesting various cavity- and open-nesting birds in all life stages [25,44]. Adult peak activity occurs from October to February [45], partially aligning with our observed October peak (Figure 2). *Ixodes ricinus* accounted for 2.6% of specimens. Although ungulates are primary hosts, it also parasitizes birds [46,47] and 18 bat species in Europe [37,48], mainly large-bodied, ground-hunting bats [37]. In this study, *I. ricinus* was recorded only in caves of northwestern Croatia (Varaždin, Croatian Zagorje: Ravna Gora, Strahinjčica), indicating that subterranean habitats are unsuitable for this exophilic species. The first Croatian subterranean record was from a cave on Mljet [19]. One adult *H. marginatum* was collected from Biokovo caves (Dalmatia), while its first subterranean record in Croatia was from Istria [19]. This species is widespread along the Adriatic coast from Pula to Dubrovnik [36,49]. Larvae and nymphs have been collected from hedgehogs [40], adults mainly from horses (*Equus caballus*), cows (*Bos taurus*), and sheep (*Ovis aries*) [36,49]. *H. marginatum* prefers high temperatures with low precipitation and humidity [50], whereas caves present opposite conditions, explaining its sporadic subterranean occurrence. Five ticks could not be identified to species. Three resemble *Ixodes lividus* Koch, 1844, an ornithophilic tick primarily associated with hole-nesting birds such as sand martins (*Riparia riparia*), and occasionally found in burrows [27,44]. While rarely recorded in caves, sporadic findings exist on bats in the UK, Germany, and the Netherlands [37]. DNA barcoding is required to confirm species identity. Similarly, the remaining two specimens resemble *Haemaphysalis erinacei* Pavesi, 1884, but species identification remains unconfirmed.

## 5. Conclusions

This study recorded seven hard tick (Ixodidae) taxa in subterranean habitats of 138 caves across 13 Croatian regions (Table 1, Figure 1). Four previously documented species were confirmed, while *I. frontalis* was recorded in subterranean habitats in Croatia for the first time. The genus *Ixodes* was the most abundant, represented by five taxa, whereas *Haemaphysalis* and *Hyalomma* were represented by a single taxon each. Two endophilic species, *I. vespertilionis* and *I. hexagonus*, dominated the sample, accounting for 91.9%, followed by the ornithophilic *I. frontalis* (3.3%). The remaining four taxa collectively accounted for 4.7%. Only *I. vespertilionis* was recorded throughout all 12 months; *I. hexagonus* appeared in nine months, *I. frontalis* in four months, and the other taxa over shorter periods. The presence of hard ticks in Dinaric subterranean habitats, reflecting their host specificity, can serve as indicators of vertebrate fauna in caves. The most abundant species, *I. vespertilionis*, indicates bat (Chiroptera) presence, particularly four primary hosts: greater mouse-eared bat (*M. myotis*) and three *Rhinolophus* species—the Mediterranean horseshoe bat (*R. euryale*), greater horseshoe bat (*R. ferrumequinum*), and lesser horseshoe bat (*R. hipposideros*). Linking individual bats or bat colonies to tick records in the same caves could inform integrated ecological analyses. Similarly, the second most abundant species, *I. hexagonus*, may indicate the presence of Mustelidae (e.g., martens, *Martes* spp.), Canidae, and occasionally bats such as *M. myotis* and *R. ferrumequinum*. Individual tick specimens collected from caves can also be tested for zoonotic pathogens, as they may serve as potential vectors between bats and non-bat hosts [51].

## Figures and Tables

**Table 1:** Hard tick (Ixodidae) taxa, life stages, and number of specimens recorded.

Taxa	Females	Males	Nymphs	∑	%
*Ixodes vespertilionis* Koch, 1844	30	175	17	222	81.0
*Ixodes hexagonus* Leach, 1815	14	16	-	30	10.9
*Ixodes frontalis* (Panzer, 1798)	-	9	-	9	3.3
*Ixodes ricinus* (Linnaeus, 1758)	1	6	-	7	2.6
*Ixodes* sp.	2	1	-	3	1.0
*Haemaphysalis* sp.	1	1	-	2	0.7
*Hyalomma marginatum* Koch, 1844	-	1	-	1	0.4
∑	48	209	17	274	99.9

**Table 2:** Diversity and abundance of hard ticks (Ixodidae) in Croatian subterranean habitats.

Region			Taxa					∑
	*Ixodes vespertilionis*	*Ixodes hexagonus*	*Ixodes frontalis*	*Ixodes ricinus*	*Ixodes* sp.	*Haemaphysalis* sp.	*Hyalomma marginatum*	
Slavonia	5	2	6	-	-	-	-	13
NW Croatia	16	11	-	7	3	-	-	37
Medvednica Mt.	9	-	-	-	-	-	-	9
Žumberak Mt.	11	1	-	-	-	-	-	12
Banovina	6	-	-	-	-	-	-	6
Pokuplje	41	11	-	-	-	-	-	52
Kordun	24	2	-	-	-	-	-	26
Lika	27	-	-	-	-	-	-	27
Istria	24	-	1	-	-	-	-	25
Kvarner Bay	8	-	-	-	-	-	-	8
Velebit Mt.	5	1	-	-	-	-	-	6
Pounje	1	-	-	-	-	-	-	1
Dalmatia	45	2	2	-	-	2	1	52
∑	222	30	9	7	3	2	1	274

Legend: Mt. (Mountain).

## Data Availability

The original data presented in the study are openly available at this article.

## Appendix A. Subterranean Localities Studied in Croatia, 1993–2024

**Table A1:** List of studied localities with UTM marks and geographic coordinates.

Ord. Nr.	Region/Locality/Village/Protected Area/Mountain/Municipality/or City/Island	UTM Mark	Geographic Coordinates
	Pannonian Range		
	Slavonia		
1.	Uviraljka, Velika, Papuk NP, Velika	YL 03	45°31′07″ N, 17°37′00″ E
2.	Jazavčeva špiljica, Kovačica, Jankovac, Papuk NP, Čačinci	YL 14	45°36′16″ N, 17°41′33″ E
3.	Jančikin guz, Jankovac, Papuk NP, Čačinci	YL 14	45°31′16″ N, 17°41′02″ E
4.	Jama na mrežarima, Mrežarski rust, Jankovac, Papuk NP, Čačinci	YL 14	45°30′50″ N, 17°42′30″ E
5.	Suhodolska jama, Suhodol, Jankovac, Papuk NP, Čačinci	YL 14	45°31′55″ N, 17°35′34″ E
6.	Špilja papučkog pračovjeka, Sokolina, Jankovac, Papuk NP, Čačinci	YL 14	45°32′13″ N, 17°37′22″ E
7.	Jelenova špilja, Sokolina, Jankovac, Papuk NP, Čačinci	YL 14	45°32′13″ N, 17°37′22″ E
8	Sokolina špilja, Gornji Vrhovci, Papuk NP, Čačinci	XL 93	45°28′26″ N, 17°34′08″ E
9.	Brunina špilja, Jovanovica, Papuk NP, Voćin	XL 95	45°37′11″ N, 17°32′41″ E
	Supradinaric Range		
	NW Croatia (Croatian Zagorje area)		
10.	Žutnica, Strahinjščica Mt., Krapina	WM 61	46°11′42″ N, 15°52′41″ E
11.	Rana Peć, Jelavica, Žutnica, Strahinjščica Mt., Krapina	WM 61	46°11′50″ N, 15°53′37″ E
12.	Vilska Luknja, Goleš, Zagora, Strahinjščica Mt., Radoboj	WM 71	46°09′56″ N, 15°52′15″ E
	NW Croatia (Varaždin area)		
13.	Vihra špiljica, potok Kamenica (Filićev dom), Kameničko Podgorje, Ravna Gora Mt., Lepoglava	WM 81	46°15′25″ N, 16°00′58″ E
14.	Jelovec jama, Cimer plac, Ravna Gora Mt., Lepoglava	WM 81	46°12′38″ N, 16°02′08″ E
15.	Zdenec pri Ciglaru, Ciglari, Ravna Gora Mt., Klenovnik	WM 82	46°16′13″ N, 16°04′37″ E
16.	Cerjanska špilja, Cerjani, Ravna Gora Mt., Klenovnik	WM 82	46°16′31″ N, 16°03′36″ E
17.	Špilja u Bukevju, Bukevje, Ivanščica Mt., Ivanec	WM 82	46°13′23″ N, 16°07′12″ E
	Medvednica Mountain		
18.	Veternica, Veroško rebro, Gornji Stenjevec, Medvednica Mt., Zagreb	WL 77	45°50′29″ N, 15°52′25″ E
19.	Židovske jame, Medvednica Mt., Gornja Stubica	WL 79	45°58′35″ N, 16°01′03″ E
	Adriatic & Dinaric Range		
	Žumberak Mountain		
20.	Obreški Grič, Obrež Vivodinski, Žumberak Mt., Ozalj	WL 35	45°46′11″ N, 15°26′32″ E
21.	Milićka jama; Kuljaji, Žumberak Mt., Ozalj	WL 35	45°43′10″ N, 15°18′30″ E
22.	Rakička špilja, Pilatovci, Žumberak Mt., Ozalj	WL 35	45°42′14″ N, 15°18′16″ E
23.	Jamina, Bučari, Donji Oštrc, Žumberak Mt.	WL 35	45°42′00″ N, 15°25′59″ E
24.	Ponor Vrulje, Cerovica, Žumberak Mt., Samobor	WL 35	45°48′34″ N, 15°28′53″ E
25.	Dolača, Drašči vrh, Žumberak Mt.	WL 36	45°45′00″ N, 15°28′59″ E
26.	Rogovac, Žumberak Mt.	WL 37	45°44′28″ N, 15°36′13″ E
27.	Provala, Sošice, Žumberak Mt.	WL 37	45°45′00″ N, 15°22′59″ E
28.	Bedara, Tihočaj, Žumberak Mt., Samobor	WL 57	45°45′14″ N, 15°33′01″ E
	Pokuplje		
29.	Gugečka špilja, Gornje Dubrave, Ogulin	WL 11	45°18′29″ N, 15°18′18″ E
30.	Ledenica u Čazinki, Donje Dubrave, Ogulin	WL 11	45°19′01″ N, 15°22′01″ E
31.	Vrelić špilja, Donje Dubrave, Ogulin	WL 11	45°19′01″ N, 15°22′01″ E
32.	Sušica ponor, Rebića Glavica, Donje Dubrave, Ogulin	WL 11	45°19′01″ N, 15°22′01″ E
33.	Kuštrovka, Popovo Selo, Trošmarija	WL 21	45°18′50″ N, 15°15′18″ E
34.	Danetska špilja, Dani, Bosiljevo	WL 22	45°21′18″ N, 15°19′37″ E
35.	Jankonka jama, Hrsine, Bosiljevo	WL 22	45°24′22″ N, 15°16′05″ E
36.	Ledenica u Špeharima, Špehari, Bosiljevo	WL 22	45°22′19″ N, 15°18′40″ E
37.	Dragina peć na Dobri, Gabrk, Podumol, Bosiljevo	WL 22	45°21′07″ N, 15°17′17″ E
38.	Jama u kanjonu Dobre ispod Gabrka, Podumol, Bosiljevo	WL 22	45°21′07″ N, 15°17′17″ E
39.	Špilja kod Podumolskog mlina, Podumol, Bosiljevo	WL 22	45°21′29″ N, 15°18′07″ E
40.	Vodena jama, Kolići, Generalski Stol	WL 22	45°20′30″ N, 15°22′32″ E
41.	Paveća špilja, Soline, Generalski Stol	WL 22	45°20′30″ N, 15°22′32″ E
42.	Lipa na Protulipi, Lipa, Generalski Stol	WL 22	45°24′36″ N, 15°23′13″ E
43.	Zvonečka II, brdo Lipnik Griče Gornje, Ribnik	WL 24	45°32′06″ N, 15°22′52″ E
44.	Jama Đot, brdo Lipnik, Ribnik	WL 24	45°34′12″ N, 15°20′60″ E
45.	Pivnica jama, Sela Žakanjska, Žakanje	WL 35	45°36′25″ N, 15°20′36″ E
46.	Hrenov Grič, Kamanje	WL 35	45°37′48″ N, 15°23′24″ E
47.	Kozjača špilja, Kamanje	WL 35	45°37′48″ N, 15°23′24″ E
48	Vrlovka špilja, Kamanje	WL 35	45°36′28″ N, 15°24′14″ E
	Banovina		
49.	Bajića špilja 1,3, Pecka, Perna, Topusko	WL 61	45°16′14″ N, 15°51′14″ E
50.	Velika špilja, Perna, Petrova Gora Mt., Topusko	WL 61	45°17′15″ N, 15°52′10″ E
51.	Gradusa špilja, Sjeverovac, Sunja	XL 22	45°20′18″ N, 16°26′36″ E
	Kordun		
52.	Rebička špilja, kanjon Tounjčice, Tounj	WL 30	45°15′00″ N, 15°19′48″ E
53.	Tounjčica špilja, Tounj	WL 30	45°15′00″ N, 15°19′48″ E
54.	Jama Mandelaja, Oštarije, Josipdol	WL 20	45°13′35″ N, 15°17′56″ E
55.	Markova špilja, Mateško Selo, Generalski Stol	WL 31	45°19′34″ N, 15°25′41″ E
56.	Matešićeva špilja, Mateško selo, Generalski Stol	WL 31	45°19′34″ N, 15°25′41″ E
57.	Mikića jama, Perjasica, Barilović	WL 31	45°17′35″ N, 15°27′54″ E
58.	Jama u Steljnici, Lučica, Siča, Barilović	WL 32	45°20′42″ N, 15°28′59″ E
59.	Vodena špilja, Siča, Barilović	WL 32	45°21′22″ N, 15°28′18″ E
60.	Špilja Gvozdenica, Krnjak	WL 42	45°20′14″ N, 15°36′30″ E
61.	Bezdanica kod Barilovića, Barilović	WL 42	45°22′48″ N, 15°32′24″ E
62.	Jopićeva špilja, Krnjak	WL 42	45°20′14″ N, 15°36′30″ E
63.	Jama pod Debelom glavom, Hrvatski Blagaj, Slunj	WL 40	45°12′55″ N, 15°32′25″ E
64.	Kutarčeva špilja, kanjon Korane, Slunj	WK 49	45°06′54″ N, 15°35′08″ E
65.	Jama 4 psa, Gnojnice, Cetingrad	WL 50	45°07′52″ N, 15°40′16″ E
66.	Vukovićeva špilja, Gnojnice, Cetingrad	WL 50	45°07′52″ N, 15°40′16″ E
67.	Špilja ispod Drežnik grada, Drežnik Grad, Rakovica	WK 57	44°56′53″ N, 15°40′23″ E
68.	Adios špilja, Kordunski Ljeskovac, Rakovica	WK 58	45°00′15″ N, 15°46′01″ E
69.	Kojina jama, Mašvina, Rakovica	WK 58	45°01′16″ N, 15°42′40″ E
70.	Baraćeva gornja špilja, Nova Kršlja, Rakovica	WK 58	44°59′06″ N, 15°46′06″ E
	Lika		
71.	Siničić špilja, Brinje	WK 08	44°59′54″ N, 15°07′45″ E
72.	Bobića pećina, Mlakva, Donji Kosinj, Perušić	WK 25	44°41′58″ N, 15°17′18″ E
73.	Čardačina jama, Mlakva, Poljane, Donji Kosinj, Perušić	WK 25	44°41′58″ N, 15°17′18″ E
74.	Horvatova špilja, HE Sklope, Donji Kosinj, Perušić	WK 25	44°46′30″ N, 15°11′24″ E
75.	Mramornjača, Petranović Draga, Donji Kosinj, Perušić	WK 25	44°09′33″ N, 15°20′43″ E
76.	Pećina u Čakovcu, Mlakva, Bobići, Donji Kosinj, Perušić	WK 25	44°41′58″ N, 15°17′18″ E
77.	Šojića pećina, Podjelar, Gornji Kosinj, Perušić	WK 25	44°42′25″ N, 15°15′38″ E
78.	Amidžina pećina, PP Grabovača, Perušić	WK 34	44°38′15″ N, 15°22′16″ E
79.	Sitvukova pećina, Sitvuk, PP Grabovača, Perušić	WK 34	44°38′15″ N, 15°22′16″ E
80.	Medina pećina, PP Grabovača, Perušić	WK 34	44°38′15″ N, 15°22′16″ E
81.	Rogić špilja, Klanac, Gospić	WK 23	44°38′04″ N, 15°17′53″ E
82.	Špilja 2 u kanjonu Jadove, Lovinac	WK 51	44°23′24″ N, 15°40′48″ E
83.	Šupljara špilja, jezero Kozjak, Plitvice Lakes NP, Korenica	WK 55	44°52′48″ N, 15°37′08″ E
84.	Špilja Milke Trnine, jezero Kozjak, Plitvice Lakes NP, Korenica	WK 55	44°52′48″ N, 15°37′08″ E
85.	Mračna pećina, kanjon Korane, Plitvice Lakes NP, Korenica	WK 55	44°52′48″ N, 15°37′08″ E
86.	Mračnjača špilja, Plitvice Lakes NP, Korenica	WK 55	44°52′48″ N, 15°37′08″ E
87.	Jama na Vršiću, Kuselj, Plitvice Lakes NP, Korenica	WK 55	44°52′48″ N, 15°37′08″ E
	Istria		
88.	Rabakova špilja, Ročko polje, Roč, Buzet	VL 22	45°22′10″ N, 14°04′44″ E
89.	Radota jama, Vodice, Jelovice, Ćićarija Mt., Lanišće	VL 23	45°29′03″ N, 14°03′14″ E
90.	Pećina pod Stržen, Stržen, Brgudac, Ćićarija Mt., Lanišće	VL 32	45°22′52″ N, 14°08′38″ E
91.	Jama kod Raspadalice, Ćićarija Mt., Lanišće	VL 32	45°24′00″ N, 14°06′58″ E
92.	Klanjčeva peć, Brest pod Učkom, Učka Mt., Lupoglav	VL 32	45°19′55″ N, 14°09′21″ E
93.	Jama pod Križ, Semići, Učka Mt., Lupoglav	VL 32	45°22′08″ N, 14°06′40″ E
94.	Grnjača špilja, Lovranska Draga, Grnjač, Učka Mt., Lovran	VL 41	45°16′42″ N, 14°14′35″ E
95.	Špilja kod Premanture, Premantura, Medulin	VK 16	44°48′04″ N, 13°54′32″ E
	Kvarner Bay		
96.	Čampari jama, Petrićevi, Cres, Cres Island	VK 57	44°51′54″ N, 14°26′41″ E
97.	Galatkovićeva jama, Krasica, Bakar	VL 61	45°18′39″ N, 13°33′23″ E
98.	Špilja Biserujka, Čižići, Rudine, Dobrinj, Krk Island	VK 69	45°11′14″ N, 14°36′35″ E
99.	Špilja iznad Jamine Drage, Lopar, Rab Island	VK 76	44°49′52″ N, 14°43′48″ E
100.	Jamice Plogar, Mundanije, Rab, Rab Island	VK 85	44°46′16″ N, 14°45′45″ E
101.	Zagorska peć, Novi Vinodolski	VK 98	45°08′41″ N, 14°47′20″ E
	Velebit Mountain		
102.	Lužina špilja, Mali Vaganac, Paklenica NP, Južni Velebit, Starigrad	WK 32	44°19′19″ N, 15°26′52″ E
103.	Manita peć, Velika Paklenica, Paklenica NP, Južni Velebit, Starigrad	WK 31	44°18′51″ N, 15°28′30″ E
104.	Topla pećina, Kaštel Žegarski, Južni Velebit, Jasenice	WJ 68	44°09′18″ N, 15°51′29″ E
105.	Plitka peć, Plitki dolovi, Čabrići Gornji, Kaštel Žegarski, Velebit NP, Obrovac	WJ 68	44°17′30″ N, 15°55′40″ E
106.	Kusa, Manastirska luka, Krupa, Velebit NP, Obrovac	WJ 79	44°11′46″ N, 15°54′20″ E
	Pounje		
107.	Rastovača špilja, kanjon Slap, Srb, Plješivica Mt., Gračac	WK 73	44°22′11″ N, 16°07′30″ E
	Dalmatia		
108.	Golubnjača špilja, Žegar, Bukovica	WJ 68	44°09′17″ N, 15°51′32″ E
109.	Rudnik Jukići, Razvođe, Oklaj, Promina	WJ 86	43°55′44″ N, 16°06′28″ E
110.	Krčić špilja, Topoljski buk, Knin	WJ 97	44°03′09″ N, 16°13′38″ E
111.	Rudelića špilja, Vukovići, Vrlika	XJ 16	43°54′32″ N, 16°24′00″ E
112.	Gospodska špilja, Četnici, Vrlika	XJ 17	43°54′32″ N, 16°24′00″ E
113.	Vodena peća, Bajagić, Sinj	XJ 34	43°45′31″ N, 16°39′58″ E
114.	Sedrena špilja iza mlina, Bilušića buk, NP Krka, Šibenik	WJ 86	44°00′47″ N, 16°04′07″ E
115	Stražbenica, Vrpolje, Šibenik	WJ 83	43°40′31″ N, 16°00′41″ E
116.	Trojama, Mosor Mt., Dugopolje	XJ 22	43°34′56″ N, 16°35′56″ E
117.	Vranjača, Kotlenice, Mosor Mt., Dugopolje	XJ 32	43°34′01″ N, 16°38′43″ E
118.	Jama pod Osojem, Nevistine stine, Donja Brela, Biokovo Mt., Brela	XJ 50	43°22′07″ N, 16°55′48″ E
119.	Drinova 2, Bartulovići, Biokovo Mt., Brela	XJ 50	43°20′31″ N, 17°03′12″ E
120.	Mala jama u Bratušu, Bratuš, Biokovo Mt., Baška Voda	XJ 50	43°19′41″ N, 16°58′37″ E
121.	Krjava 2, Velo brdo, Biokovo Mt., Makarska	XH 69	43°19′12″ N, 17°01′16″ E
122.	Jama za Supinom, Supin, Gornje Tučepi, Biokovo Mt., Tučepi	XH 69	43°15′05″ N, 17°06′38″ E
123.	Baba špilja, Štedovac, Gornje Tučepi, Biokovo Mt., Tučepi	XH 69	43°17′17″ N, 17°05′23″ E
124.	Velika Prdaljka, Alerići, Poljica Kozička, Vrgorac	XH 98	43°18′22″ N, 17°15′42″ E
125.	Velika špilja kod Antunovića, Kozica, Šibenik Mt., Vrgorac,	XH 98	43°16′05″ N, 17°12′33″ E
126	Rudnik od Vore, Kostirna, Vis, Vis Island	WH 96	43°01′23″ N, 17°06′0″ E
127.	Jezero na Gatuli, Komiža, Biševo Island	WH 95	43°02′40″ N, 16°05′31″ E
	Dalmatia (Dubrovnik area)		
128.	Pišurka, Korčula, Korčula Island	XH 75	42°57′30″ N, 17°08′03″ E
129.	Jakasova špilja, Žrnovo, Korčula, Korčula Island	XH 75	42°56′34″ N, 17°06′37″ E
130.	Samograd špilja, Račišće, Korčula, Korčula Island	XH 75	42°58′23″ N, 17°01′11″ E
131.	Crno jezero, Ponikve, Ston, Pelješac	YH 14	42°50′20″ N, 17°38′01″ E
132.	Medviđa Ropa, Skrivena Luka, Lastovo, Lastovo Island	XH 53	42°44′13″ N, 16°53′10″ E
133.	Jama u Zadubravici, Riđica, Dubrovačko primorje	YH 34	42°47′06″ N, 17°53′24″ E
134.	Špilja u ogradi, Trnova, Dubrovačko primorje	YH 34	42°48′36″ N, 17°51′54″ E
135.	Špilja pod Gromačkom vlakom, Gromača, Dubrovačko primorje	BN 53	42°43′35″ N, 18°00′40″ E
136.	Špilja na vrh Krčevina, Orašac, Gromača, Dubrovačko primorje	BN 53	42°42′07″ N, 18°00′25″ E
137.	Banova ljut, Ljubač, Dubrovačko primorje	BN 53	42°43′21″ N, 18°01′27″ E
138.	Jama Bezdan, Komaji, Konavle	BN 71	42°32′17″ N, 18°18′30″ E

Legend: Ord. Nr. (Ordinal number), NP (Nature or National Park), UTM (Universal Transverse Mercator grid), YL, XL, WM etc. (field label 100 × 100 km).

## Appendix B. Hard Tick (Ixodidae) Records from Subterranean Habitats in Croatia, 1993–2024

**Table A2:** List of recorded hard ticks (Ixodidae) in Croatian subterranean habitats.

1. *Haemaphysalis* sp. Region: Dalmatia (Dubrovnik area) County: Dubrovnik-Neretva Records: Špilja na vrh Krčevina, Orašac-Gromača, Dubrovnik 21 June 2009 K. Miculinić leg. (1♂), 9 October 2009 H. Cvitanović leg. (1♀), (ADIPA).
2. *Hyalomma marginatum* Koch, 1844 Region: Dalmatia (including the Dubrovnik area) County: Split-Dalmatia Records: Jama za Supinom, Supin, Gornje Tučepi, Biokovo Mt., Tučepi 15 August 2012 R. Ozimec leg. (1♂), (ADIPA).
3. *Ixodes frontalis* (Panzer, 1798) Region: Slavonia County: Virovitica-Podravina, Požega-Slavonia Records: Jančikin guz, Jankovac, Papuk NP, Čačinci 25 October 2008, R, Ozimec, H. Cvitanović leg. (1♂); Sokolina špilja, Gornji Vrhovci, Papuk NP, Čačinci 25 October 2008, N. Raguž leg. (2♂); Jazavčeva špiljica, Kovačica, Jankovac, Papuk NP, Čačinci 22 September 2010, M. Lukić leg. (1♂); Jelenova špilja, Sokolina, Jankovac, Papuk NP, Čačinci 13 August 2015, N. Kuharić leg. (1♂); Špilja papučkog pračovjeka, Sokolina, Jankovac, Papuk NP, Čačinci 14 August 2015, N. Kuharić leg. (1♂) (ADIPA). Region: Istria County: Istria Records: Klanjčeva peć, Brest pod Učkom, Učka Mt., Lupoglav 6 Novemeber 2006 H. Cvitanović leg. (1♂), (ADIPA). Region: Dalmatia County: Split-Dalmatia Records: Mala jama u Bratušu, Bratuš, Biokovo Mt., Baška Voda 11 October 2018 T. Tursić leg. (2♂), (ADIPA).
4. *Ixodes hexagonus* Leach, 1815 Region: Slavonia County: Virovitica-Podravina, Požega-Slavonia Records: Brunina špilja, Jovanovica, Papuk NP, Voćin 22 September 2010, A. Komerički leg. (1♀); Sokolina špilja, Gornji Vrhovci, Papuk NP, Čačinci 19 July 2024, M. Lukić leg. (1♀), (ADIPA). Region: NW Croatia (Croatian Zagorje and Varaždin area) County: Varaždin, Krapina-Zagorje Records: Zdenac pri Ciglaru, Ciglari, Ravna Gora Mt., Klenovnik 15 May 2006, M. Pavlek leg. (2♂), H. Cvitanović leg. (1♀), 26 May 2007, F. Kljaković-Gašpić leg. (2♂), H. Cvitanović leg (1♀), 18 October 2008, E. Domina leg. (1♀, 2♂); Špilja u Bukevju, Bukevje, Ivanščica Mt., Ivanec 18 October 2008, R. Ozimec leg. (1♂); Rana peć, Jelavica, Žutnica, Strahinjčica Mt., Krapina 10 November 2009, R. Ozimec leg. (1♀), (ADIPA). Region: Žumberak County: Karlovac Records: Obreški Grič, Obrež Vivodinski, Ozalj 16 November 2002, R. Ozimec leg. (1♀), (ADIPA). Region: Pokuplje County: Karlovac Records: Ledenica u Čazinki, Donje Dubrave, Ogulin 15 June 2008 leg. J. Bedek (1♂); Gugečka špilja, Gornje Dubrave, Ogulin 17 June 2008 leg. R. Ozimec (1♀); Dragina peć na Dobri Grabrk, Podumol, Bosiljevo 18 June 2008 H. Cvitanović leg. (1♂); Paveća špilja, Soline, Generalski Stol 18 June 2008 R. Ozimec leg. (1♀); Kuštrovka, Popovo Selo, Trošmarija 27 August 2008 R. Ozimec leg. (1♀); Danetska špilja, Dani, Bosiljevo 12 May 2009 A. Komerički leg. (1♂); Sušica ponor, Rebića Glavica, Donje Dubrave, Ogulin 14 May 2009 leg. A. Komerički (1♂); Pivnica jama, Sela Žakanjska, Žakanje 5 June 2009 H. Cvitanović leg. (1♂); Kozjača špilja, Kamanje 7 June 2009 H. Cvitanović leg. (1♂), K. Miculinić leg. (1♂); Hrenov Grič, Kamanje 1 February 2015, H. Cvitanović leg. (1♀), (ADIPA). Region: Kordun County: Karlovac Records: Špilja ispod Drežnik grada, Drežnik Grad, Rakovica 28 April 2004 I. Franjković leg. (1♂); Rebička špilja, kanjon Tounjčice, Tounj 27 August 2004 J. Bedek and H. Cvitanović leg (1♂), (ADIPA). Region: Velebit Mt. County: Zadar Records: Topla pećina, Kaštel Žegarski, Južni Velebit, Jasenice 25 February 2008 R. Ozimec leg. (1♀), (ADIPA). Region: Dalmatia (including the Dubrovnik area) County: Šibenik-Knin Rudnik Jukići, Razvođe, Oklaj, Promina 9 July 2020 R. Ozimec leg. (1♀), (ADIPA). County: Dubrovnik-Neretva Records: Špilja u ogradi, Trnova, Dubrovačko primorje 16 October 2011 R. Ozimec leg. (1♀), (ADIPA).
5. *Ixodes* sp. Region: NW Croatia (Varaždin area) County: Varaždin Records: Zdenac pri Ciglaru, Ciglari, Ravna Gora Mt., Klenovnik 15 May 2006, H. Cvitanović leg. (1♀), 26 May 2007, F. Kljaković-Gašpić leg. (1♂), 18 October 2008, F. Kljaković-Gašpić leg. (1♀), (ADIPA).
6. *Ixodes ricinus* (Linnaeus, 1758) Region: NW Croatia (Croatian Zagorje and Varaždin area) County: Varaždin, Krapina-Zagorje Records: Zdenac pri Ciglaru, Ciglari, Ravna Gora Mt., Klenovnik 26 May 2007, F. Kljaković-Gašpić leg. (5♂); Vilska Luknja, Goleš, Zagora, Strahinjčica Mt., Radoboj 16 May 2009, R. Ozimec leg. (1♂); Rana Peć, Jelavica, Žutnica, Strahinjčica Mt., Krapina 16 May 2009, A. Komerički leg. (1♀), (ADIPA).
7. *Ixodes vespertilionis* Koch, 1844 Region: Slavonia County: Virovitica-Podravina, Požega-Slavonia Records: Ponor Uviraljka, Papuk NP, Velika 19 July 2004, H. Bilandžija leg. (1♂), 21 September 2010, M. Lukić leg. (1♂); Suhodolska Jama, Suhodol, Jankovac, Papuk NP, Čačinci 24 October 2008, R. Ozimec leg. (2♂); Jama na mrežarima, Mrežarski rust, Jankovac, Papuk NP, Čačinci 10 August 2015, H. Višić leg. (1♀), (ADIPA). Region: NW Croatia (Croatian Zagorje and Varaždin area) County: Varaždin, Krapina-Zagorje Records: Cerjanska špilja, Cerjani, Ravna Gora Mt., Klenovnik 9 January 2002 R. Ozimec leg. (2♂), 15 May 2006 M. Pavlek leg. (3♂, 1n), R. Ozimec (1♀); Vihra špiljica, potok Kamenica, (Filićev dom), Kameničko Podgorje, Ravna Gora Mt., Lepoglava 13 May 2006, H. Cvitanović leg. (1♂); Zdenac pri Ciglaru, Ciglari, Ravna Gora Mt., Klenovnik 26 May 2007, H. Cvitanović leg. (1♂), 18 October 2008 E. Domina leg. (1n); Jelovec jama, Cimerplac, Ravna Gora Mt., Lepoglava 28 May 2007, F. Kljaković-Gašpić leg. (1♀); Vilska Luknja, Goleš, Zagora, Strahinjščica Mt., Radoboj 16 May 2009, R. Ozimec leg. (2♀,2n), A. Komerički leg. (1n), (ADIPA). Region: Medvednica Mt. County: Krapina-Zagorje, Zagreb Records: Židovske jame, Medvednica Mt., Gornja Stubica 23 April 2005, R. Ozimec leg. (2♂); Veternica, Veroško rebro, Gornji Stenjevec, Medvednica Mt., Zagreb 31 May 2009, D. Bakšić leg. (1♂), 25 November 2012, R. Ozimec leg. (1♂), 30 August 2013, R. Ozimec leg. (2♂), 1 December 2016, R. Ozimec leg. (2♂), -, M. Jagić (1♂), (ADIPA). Region: Žumberak County: Zagreb, Karlovac Records: Špilja Provala, Sošice, Žumberak 2 February 1997, R. Ozimec leg. (1♂); Rogovac, Žumberak 18 January 1998, T. R leg. (1♂); Jamina Bučari, Donji Oštrc, Žumberak 19 April 1998, R. Ozimec leg. (1♂), 17 October 1999, R. Ozimec leg. (1♂), 2 November 2002, R. Ozimec and T. Rubinić leg. (1♂); Špilja Rakička, Pilatovci, Žumberak, Ozalj 20 September 2002, R. Ozimec and T. Rubinić leg. (1♀, 1n); Ponor Vrulje, Cerovica, Žumberak, Samobor 3 November 2002. R. Ozimec and T. Rubinić leg. (1♂); Jama Milićka, Kuljaji, Žumberak, Ozalj 26 April 2003, R. Ozimec leg. (1♀); Špilja, Dolača, Drašči vrh, Žumberak 12 December 2004, M. Pavlek leg. (1♂); Bedara, Tihočaj, Žumberak, Samobor 18 December 2005 M. Pavlek leg. (1♂), (ADIPA). Region: Pokuplje County: Karlovac Records: Pivnica jama, Sela Žakanjska, Žakanje -, 1993, H. Cvitanović leg. (1♂), 12 July 1998, R. Ozimec leg. (2♂), 18 March 2009, H. Cvitanović leg. (1♂), M. Lukić leg. (2♂), 5 June 2009, K. Miculinić leg. (1♂), 13 August 2017, D. Basara leg. (1♀), D. Belavić leg. (1♂); Jankonka jama, Hrsine, Bosiljevo 16 January 1999 R. Ozimec leg. (1♀), 2 April 2000 R. Ozimec and H. Cvitanović leg. (1♂); Jama u kanjonu Dobre ispod Gabrka, Podumol, Bosiljevo 24 June 2008 H. Cvitanović leg. (1♂), 26 August 2008 M. Pavlek leg. (1♂), M. Lukić leg. (1♂), 8 December 2008 K. Miculinić leg. (1♂), 12 May 2009 M. Lukić leg. (1♂), A. Kirin leg. (1♂), 6 September 2009 H. Cvitanović leg. (1♂), M. Lukić leg. (1♂); Dragina peć na Dobri Gabrk, Podumol, Bosiljevo 18 June 2008 H. Cvitanović leg. (1♀, 1♂), 8 December 2008 H. Cvitanović leg. (1♂), 3 October 2009 R. Baković leg. (2♂); Lipa na Protulipi, Lipa, Generalski Stol 21 June 2008 R. Baković leg. (1♂); Vodena jama, Kolići, Generalski Stol 9 May 2009 leg. A. Kirin (1♂), 10 May 2009 leg. M. Lukić (1♂); Špilja kod Podumolskog mlina, Podumol, Bosiljevo 14 May 2009 A. Krin leg. (2♂); Kuštrovka, Popovo Selo, Trošmarija 14 February 2009 M. Pavlek leg. (2♂); Zvonečka II, brdo Lipnik, Griče Gornje, Ribnik 19 March 2009, H. Cvitanović leg. (1♂), -, H. Cvitanović leg. (2♂); Jama Đot, Lipnik, Ribnik 20 March 2009 leg. J. Bedek (1♀); Vrelić špilja, Donje Dubrave, Ogulin 14 May 2009 leg. J. Bedek (1♂); Ledenica u Špeharima, Špehari, Bosiljevo 24 May 2009 M. Lukić leg. (1♂); Hrenov Grič, Kamanje 1 February 2015, H. Cvitanović leg. (1n); Vrlovka špilja, Kamanje 22 December 2015, R. Ozimec leg. (2♂), 21 July 2016, H. Cvitanović leg. (1♀), (ADIPA). Region: Banovina County: Sisak-Moslavina Records: Bajića špilja 1, Pecka, Perna, Topusko 26 February 2009 N. Gruborović leg. (1♂, 1♀), R. Ozimec and P. Rade leg. (1♀), 5 June 2002 K. Badovinac leg. (1♂), 7 March 2009 H. Cvitanović and N. Matoš leg. (1♂); Gradusa špilja, Sjeverovac, Sunja 6 March 2009 R. Ozimec leg. (1♂), (ADIPA). Region: Kordun County: Karlovac Records: Špilja Tounjčica, Tounj 14 April 1997 H. Cvitanović leg (1♂), 15 February 2009 M. Pavlek leg. (1♂); Špilja Gvozdenica, Krnjak 9 December 1997 R. Ozimec leg. (1♂); Jopićeva špilja, Krnjak 21 January 1998 R. Ozimec leg. (1♂), 6 April 1999 R. Ozimec leg. (2♂); Matešićeva špilja, Mateško selo, Generalski Stol 23 April 1998 R. Ozimec leg. (2♂, 1n); Markova špilja, Mateško Selo, Generalski Stol 16 January 2000 H. Cvitanović leg. (1♂); Bezdanica kod Barilovića, Barilović 16 May 1999 H. Cvitanović leg. (1♂); Kojina jama, Mašvina, Rakovica 27 June 1999 H. Cvitanović leg. (1♂); Jama 4 psa, Gnojnice, Cetingrad -, 2001 H. Cvitanović leg. (1♂); Vukovićeva špilja, Gnojnice, Cetingrad 3 November 2001 H. Cvitanović leg. (1♂), 5 May 2002 H. Cvitanović leg. (1♀); Vodena špilja, Siča, Barilović 19 May 2002 H. Cvitanović and K. Badovinec leg. (1♂); Baraćeva gornja špilja, Nova Kršlja, Rakovica 15 June 2003 D. Hamidović leg. (1♂); Jama Mandelaja, Oštarije, Josipdol 17 April 2004 M. Lukić leg. (1♂); Jama pod Debelom glavom, Hrvatski Blagaj, Slunj 1 May 2004 M. Pavlek leg. (1♂), 11 November 2017 D. Belavić leg. (1♀); Kutarčeva špilja, kanjon Korane, Slunj 25 May 2008 J. Bedek leg. (1♂); Mikića jama, Perjasica, Barilović 24 January 2010 leg. H. Cvitanović (1♂); Jama u Steljnici, Lučica, Siča, Barilović 6 January 2014 N. Cvitanović leg. (1♂); Adios špilja, Kordunski Ljeskovac, Rakovica 1 September 2018 D. Basara leg. (1♂), (ADIPA). Region: Lika County: Lika-Senj Records: Mramornjača, Petranović Draga, Donji Kosinj, Perušić 30 May 1999 R. Ozimec leg. (1♂), 22 November 2016 R. Ozimec leg. (1♂); Špilja 2 u kanjonu Jadove, Lovinac 4 December 2002 H. Cvitanović leg. (1♀); Šupljara špilja, jezero Kozjak, Plitvice Lakes NP, Korenica 24 September 2005 M. Pavlek leg. (1♂); Špilja Milke Trnine, jezero Kozjak, Plitvice Lakes NP, Korenica 24 September 2005 R. Ozimec leg. (1♀); Mračna pećina, kanjon Korane, Plitvice Lakes NP, Korenica 24 September 2005 M. Pavlek leg. (2♂), R. Ozimec leg. (1♂), M. Lukić leg. (1♀); Sinčić špilja, Brinje 7 October 2006 M. Pavlek leg. (1♂); Rogić špilja, Klanac, Gospić 25 July 2007 M. Pavlek leg. (1♂); Mračnjača špilja, Plitvice Lakes NP, Korenica 14 July 2008 H. Cvitanović leg. (1♂); Sitvukova pećina, Sitvuk, PP Grabovača, Perušić 8 August 2014 J. Bedek leg. (1♂); Amidžina pećina, PP Grabovača, Perušić 11 May 2014 L. Kekelj leg. (1n), 11 May 2014 K. Cindrić leg. (1♂); Bobića pećina, Mlakva, Donji Kosinj, Perušić 13 April 2016 R. Ozimec leg. (2♂); Čardačina jama, Mlakva Poljane, Donji Kosinj, Perušić 26 August 2016 D. Basara leg. (1♀), 30 September 2016 D. Basara leg. (1n); Pećina u Čakovcu, Mlakva, Bobići, Donji Kosinj, Perušić 26 August 2016 H. Cvitanović leg. (1♂), 23 November 2016 R. Ozimec leg. (1♂); Horvatova špilja, HE Sklope, Donji Kosinj, Perušić 27 August 2016 G. Polić leg. (1♂), 28 September 2016 R. Ozimec leg. (1♂); Šojića pećina, Podjelar, Gornji Kosinj, Perušić 27 September 2016 R. Ozimec leg. (2♀); Medina pećina, PP Grabovača, Perušić 16 October 2017 leg. D. Basara (1♂); Jama na Vršiću, Kuselj, Plitvice Lakes NP, Korenica 17 October 2020 D. Basara leg. (1♂), (ADIPA). Region: Istria County: Istria, Primorje-Gorski Kotar Records: Jama kod Raspadalice, Ćićarija Mt. Lanišće 11 May 2002 R. Ozimec leg. (1♂); Špilja kod Premanture, Premantura, Medulin 13 July 2004 M. Pavlek leg. (1♂); Rabakova špilja, Ročko polje, Roč, Buzet 4 July 2005 M. Lukić leg. (1♂), 6 July 2010 R. Ozimec leg. (3n), 26 November 2010 S. Polak leg. (4♂), R. Ozimec leg. (1♂); Grnjača špilja, Lovranska Draga, Grnjač, Učka Mt. Lovran 24 June 2009 J. Bedek leg. (1♂), M. Lukić leg. (1♀, 1n); Pećina pod Stržen, Stržen, Brgudac, Ćićarija Mt. Lanišće 27 June 2009 J. Bedek leg. (1♂); Jama pod Križ, Semići, Učka Mt. Lupoglav 27 June 2009 M. Lukić leg. (1♂), 4 June 2010 M. Lukić leg. (1♂); Radota jama, Vodice, Jelovice, Ćićarija Mt., Lanišće 27 November 2010 S. Polak leg. (6♂), R. Ozimec leg. (1♂), (ADIPA). Region: Kvarner Bay County: Primorje–Gorski Kotar Records: Galatkovićeva jama, Krasica, Bakar 13 October 1996 G. Polić leg. (1♂); Jamice Plogar, Mundanije, Rab, Rab Island 17 May 1997 R. Ozimec leg. (2♂); Špilja iznad Jamine Drage, Lopar, Rab Island 18 May 1997 R. Ozimec leg. (1♀); Čampari jama, Petrićevi, Cres, Cres Island 8 April 2001 R. Ozimec and B. Jalžić leg. (2♂); Špilja Biserujka, Čižići, Rudine, Dobrinj, Krk Island 26 May 2009 R. Ozimec and P. Rade leg. (1♀), 17 March 2011 R. Ozimec leg. (1♂); Zagorska peć, Sjeverni Velebit, Novi Vinodolski 25 May 2018 D. Basara leg. (1♂), (ADIPA). Region: Velebit Mt. County: Zadar, Lika-Senj Records: Manita peć, Velika Paklenica, Paklenica NP, Južni Velebit, Starigrad 7 June 2006 M. Pavlek leg. (1♂); Lužina špilja, Mali Vaganac, Paklenica NP, Južni Velebit, Starigrad 9 June 2006 M. Pavlek leg. (1♂); Kusa, Manastirska luka, Krupa, Velebit PP, Obrovac 1 August 2006 R. Ozimec leg (1♀); Plitka peć, Plitki dolovi, Čabrići Gornji, Kaštel Žegarski, Velebit NP, Obrovac 2 May 2009 R. Baković leg (1♂), (ADIPA). Region: Pounje County: Zadar Records: Rastovača špilja, kanjon Slap, Srb, Plješivica Mt., Gračac 21 June 2005 J. Bedek leg. (1♂), (ADIPA). Region: Dalmatia (including the Dubrovnik area) County: Split-Dalmatia, Dubrovnik-Neretva, Zadar, Šibenik-Knin Records: Rudnik od Vore, Kostirna, Vis, Vis Island 8 January 2000 R. Ozimec leg. (2♂); Baba špilja, Štedovac, Gornje Tučepi, Biokovo Mt., Tučepi 27 April 2002 R. Ozimec leg. (2♂), 5 November 2009 H. Cvitanović leg. (1♂), 17 October 2011 D. Basara leg. (1♂); Krjava 2, Velo brdo, Biokovo Mt., Makarska 24 March 2003 R. Ozimec leg (1♂), 27 October 2006 S. Nižetić leg. (1n), 2 June 2023 D. Basara leg. (1♂); Jama pod Osojem, Nevistine stine, Donja Brela, Biokovo Mt., Brela 28 October 2003 J. Bedek leg. (1♀); Špilja pod Gromačkom vlakom, Gromača, Dubrovnik 2 November 2003 J. Bedek leg. (1♂); Rudelića špilja, Vukovići, Vrlika 22 August 2004 M. Pavlek leg. (1♂); Golubnjača špilja, Žegar, Bukovica 9 January 2005 H. Bilandžija leg. (1♂), M. Pavlek leg. (1♀), J. Bedek leg (1♂), 2 August 2006 M. Pavlek leg. (1♂), R. Ozimec leg. (1♀), 24 February 2008 H. Cvitanović leg. (1♂); Stražbenica, Vrpolje, Šibenik 29 March 2005 J. Bedek leg. (1♂); Trojama, Mosor Mt., Dugopolje 6 May 2005 R. Ozimec leg. (1♂); Gospodska špilja, Četnici, Vrlika 14 August 2005 M. Pavlek leg. (1♂); Jezero na Gatuli, Komiža, Biševo Island 2 October 2005 R. Ozimec leg. (1♂); Vodena peća, Bajagić, Sinj 4 February 2006 M. Pavlek leg. (1♂); Vranjača, Kotlenice, Mosor Mt., Dugopolje 1 August 2007 H. Bilandžija leg. (1♂), M. Lukić leg. (1♂); Medviđa Ropa, Skrivena Luka, Lastovo, Lastovo Island 3 March 2008. Manger and Drčelić leg. (1♂,1n); Jama u Zadubravici, Riđica, Dubrovnik 31 March 2008 R. Ozimec leg. (1♂); Banova ljut, Ljubač, Dubrovnik 31 October 2008 H. Cvitanović leg. (1♂), 11 April 2011 R. Ozimec leg. (1♂); Jakasova špilja, Žrnovo, Korčula, Korčula Island 16 March 2010 R. Ozimec leg. (2♀), 11 November 2019 K. Vučinić Bračić leg. (1♂); Crno jezero, Ponikve, Ston, Pelješac 14 April 2011 I. Njunjić leg. (1♂); Jama Bezdan, Komaji, Konavle 13 August 2011 M. Kajinić leg. (1n); Velika Prdaljka, Alerići, Poljica Kozička, Vrgorac, 6 November 2015 Ž. Marunčić leg. (1♂), M. Horvatin leg. (1♂); Sedrena špilja iza mlina, Bilušića buk, NP Krka, Šibenik 24 June 2018 R. Ozimec leg. (1♂); Krčić špilja, Topoljski buk, Knin 26 July 2018 R. Ozimec leg. (1♂); Pišurka, Korčula, Korčula Island 4 December 2018 R. Ozimec leg. (1♂); Samograd špilja, Račišće, Korčula, Korčula Island 7 December 2018 R. Ozimec leg. (1♂); Velika špilja kod Antunovića, Kozica, Biokovo Mt., Vrgorac 4 June 2019 R. Ozimec and I. Vuković leg. (1♂); Drinova 2, Bartulovići, Biokovo Mt., Brela, 6 June 2019 R. Ozimec and I. Vuković leg. (2♂); Jama za Supinom, Supin, Gornje Tučepi, Biokovo Mt., Tučepi 4 July 2022 D. Basara and P. Rade leg. (1♂), (ADIPA).

Legend: ♀ (female), ♂ (male).
